# Anti-invasive and antiangiogenic effects of MMI-166 on malignant glioma cells

**DOI:** 10.1186/1471-2407-10-339

**Published:** 2010-06-29

**Authors:** Hiromichi Nakabayashi, Toshio Yawata, Keiji Shimizu

**Affiliations:** 1 Department of Neurosurgery, Kochi Medical School Kochi University, Nankoku, Japan

## Abstract

**Background:**

The constitutive overexpression of matrix metalloproteinases (MMPs) is frequently observed in malignant tumours. In particular, MMP-2 and MMP-9 have been reported to be closely associated with invasion and angiogenesis in malignant gliomas. Our study aimed to evaluate the antitumour effects of MMI-166 (Nalpha-[4-(2-Phenyl-2H- tetrazole-5-yl) phenyl sulfonyl]-D-tryptophan), a third generation MMP inhibitor, on three human glioma cell lines (T98G, U87MG, and ONS12) in vitro and in vivo.

**Methods:**

The effects of MMI-166 on the gelatinolytic activity was analysed by gelatine zymography. The anti-invasive effect of MMI-166 was analysed by an in vitro invasion assay. An in vitro angiogenesis assay was also performed. In vitro growth inhibition of glioma cells by MMI-166 was determined by the MTT assay. The effect of MMI-166 on an orthotropic implantation model using athymic mice was also evaluated.

**Results:**

Gelatine zymography revealed that MMP-2 and MMP-9 activities were suppressed by MMI-166. The invasion of glioma cells was suppressed by MMI-166. The angiogenesis assay showed that MMI-166 had a suppressive effect on glioma cell-induced angiogenesis. However, MMI-166 did not suppress glioma cell proliferation in the MTT assay. In vivo, MMI-166 suppressed tumour growth in athymic mice implanted orthotropically with T98G cells and showed an inhibitory effect on tumour-induced angiogenesis and tumour growth. This is the first report of the effect of a third generation MMP inhibitor on malignant glioma cells.

**Conclusions:**

These results suggest that MMI-166 may have potentially suppressive effects on the invasion and angiogenesis of malignant gliomas.

## Background

**Figure 1:**
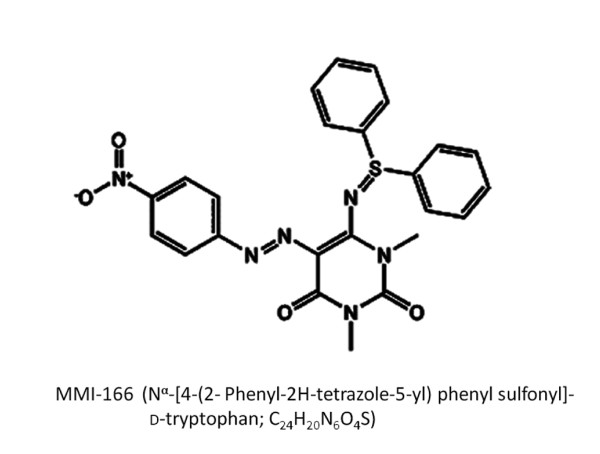
**The chemical structure of MMI-166**. MMI-166, a third generation MMP inhibitor, selectively inhibits the activity of MMP-2, -9, and -14.

Malignant gliomas are characterized by high invasive potential and strong angiogenic ability. The control of tumour invasion and angiogenesis are the key problems for the improvement of treatment results of malignant gliomas. Tumour invasion and angiogenic processes are involved in the degradation of the extracellular matrix (ECM) that surrounds tumour cells. Matrix metalloproteinases (MMPs) that degrade various ECM components are frequently expressed in malignant tumours, including gliomas, at higher levels than their benign counterparts [1,2]. MMPs are theoretically promising targets for new drugs to treat cancers. Several MMP inhibitors have been developed, and their clinical trials have begun in cancer patients [3-5]. Nevertheless, many clinical failures have made drug developers prudent, and the era of synthetic MMP inhibitors was thought to be finished. However, these failures of MMP inhibitor clinical trials in cancer were partly due to the inadvertent inhibition of MMP antitargets that are crucial for host protection. Achieving the selectivity of MMP inhibitor may validate clinical application of MMP inhibitors. It might be meaningful to test novel selective MMP inhibitors. Then, we aimed to examine the inhibitory effects of MMI-166 (N^α^-[4-(2- Phenyl-2H-tetrazole-5-yl) phenyl sulfonyl]-d-tryptophan; C_24_H_20_N_6_O_4_S) (Figure 1), a third generation MMP inhibitor, on the invasive and angiogenic processes of human malignant glioma cell lines in vitro and in vivo. MMI-166 has a selective spectrum of MMP inhibition (MMP-2, MMP-9, and MMP-14) in order to reduce side effects. The molecular weight of this inhibitor is 488.5 Da, and it is expected to cross the blood-brain barrier. This is the first report of the effect of a third generation MMP inhibitor on human malignant glioma cells.

## Methods

### Glioma cell lines and cell culture

Two human glioma cell lines (T98G and U87MG) were obtained from the American Type Culture Collection (Rockville, MD, USA). The ONS12 cell line was established from the resected tumour tissue of a 48-year-old female with glioblastoma in our hospital. These cell lines were maintained in Dulbecco's modified Eagle's medium (DMEM; Nikken Biomedical Laboratory, Kyoto, Japan) supplemented with 10% fetal bovine serum (FBS; Gibco BRL, Gaithersburg, MD, USA), penicillin (100 unit/mL), and streptomycin (100 mg/mL) at 37°C in tissue culture dishes (Asahi Techno Glass Corporation, Chiba, Japan) in a humidified incubator gassed with 5% CO_2_.

### MMI-166

MMI-166 was kindly provided by Shionogi Research Laboratory (Osaka, Japan). MMI-166 selectively inhibits the activity of MMP-2, -9, and -14 (IC50s: 0.4, 90, and 100 nmol/L, respectively) but not the activity of MMP-1, -3, or -7 (IC50s: >1,000 nmol/L) [6].

### Gelatine zymography

Gelatine substrate gel electrophoresis was performed to measure the levels of metalloproteinase activity in culture supernatants from the cell lines by using the gelatine zymography kit (Yagai Corporation, Tokyo, Japan). Glioma cell lines were seeded onto plates containing DMEM with 10% FBS. When the cells had grown to approximately 80% confluency, the medium was removed, and the cells were washed 3 times with DMEM to remove residual FBS. The cells were then cultured for 24 h in DMEM with 0.1% bovine serum albumin (BSA). After 24 h, the culture medium was collected and centrifuged twice at 800 rpm for 5 min. The supernatant (20 μL) was electrophoresed on the gel supplied with the gelatine zymography kit. The gel was washed with two types of washing buffer for 30 min each and then incubated for 30 h at 37°C in the reaction buffer. To assess the MMP inhibitory activity of MMI-166, the gels were incubated in a reaction buffer containing various concentration levels of MMI-166 (0, 0.1, 1, 10, and 100 μM). The gels were stained with Coomassie blue and then destained. The gelatinolytic activity was visualized as clear white bands against a blue background.

### Invasion assay

The invasion assay was performed using Transwell invasion chambers (BioCoat; BD Biosciences, San Jose, CA, USA). Glioma cell lines were cultured in 24-well plates. An insert was used to divide each well of the plate into lower and upper chambers. The bottom of the insert was an 8.0-μm pore size PET membrane coated with Matrigel (BD Biosciences). The lower chamber was filled with 700 μL DMEM supplemented with 0.1% BSA culture medium and human fibronectin (12.5 μg/dL, as a chemoattachment). The subconfluent cells were harvested and suspended in 500 μL DMEM supplemented with 0.1% BSA culture medium containing one of various MMI-166 concentrations (0.1-100 μM). The cells were subsequently cultured at a density of 5.0 × 10^4 ^cells/well in the upper chamber. After incubation for 23 h, the cells present on the upper surface of the filters were removed with cotton swabs. The filters were fixed in 70% ethanol and stained with Giemsa. Cells on the lower surface were counted under × 200 magnifications in five randomized field views. The number of invading cells was compared between MMI-166 and control (non-MMI-166) conditions. The invasion assay was conducted three times for every cell line.

### Angiogenesis assay

**Figure 2:**
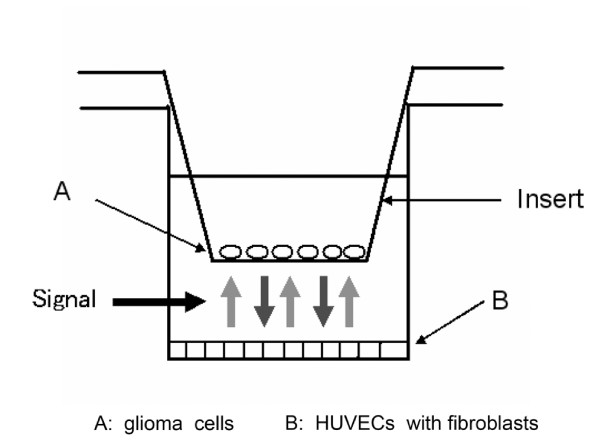
**A new method for in vitro angiogenesis assay**. The effects of MMI-166 on glioma-induced angiogenesis were determined by the newly devised research technique based on the angiogenesis kit (Kurabo, Osaka, Japan). Glioma cells were cultured in the insert plate so that soluble angiogenic factors secreted by the glioma cells could affect human umbical vein endothelial cells (HUVECs) in the lower chamber.

The effects of MMI-166 on glioma-induced angiogenesis were determined by a newly devised research technique based on the angiogenesis kit (Kurabo, Osaka, Japan) (Figure 2). Human endothelial cells and fibroblasts were incubated together according to the kit manufacturer's instructions. We also placed an insert plate with a 1.0-μm pore size PET membrane (Falcon HTS Multiwell Insert Systems, BD Biosciences) on the 24-well plate of the angiogenesis kit. Glioma cells were cultured in the insert plate so that soluble angiogenic factors secreted by the glioma cells could affect endothelial cells in the lower chamber. Once the endothelial cells had reached the early stage of lumen formation, MMI-166 was added to the culture solution at varying concentration levels (0.1-100 μM) and incubated for 10 days. MMI-166 was not added in the control condition. On day 11 of incubation, the cells were fixed in 70% ethanol, and the anti-CD31 monoclonal antibody (Kurabo) was used as the primary antibody to immunohistochemically stain the vascular lumens. The angiogenesis assay was conducted three times for every cell line.

### MTT assay

For the determination of in vitro growth inhibition of glioma cells by MMI-166, the 3-(4,5-dimethylthiazol-2-yl)-2,5-diphenyltetrazolium bromide (MTT) assay was used. Glioma cells (1 × 10^4 ^cells/well) were plated in 96-well plates in 100 mL of the culture medium. After 24 h, MMI-166 (0, 0.1, 1, 10, and 100 μM) was added to each well. After 24 and 48 h of incubation with or without MMI-166, 50 μL MTT (2 mg/mL in PBS) was added to each well at 37°C for 3 h, and MTT reduction by viable cells was measured colorimetrically at 570 nm using a Universal Microplate Reader (EL800; BioTek Instruments, Inc., Winooski, VT, USA). The MTT assay was conducted three times for every cell line.

### Effect of MMI-166 on orthotropic implantation

Athymic female mice (BALB/c *nu/nu*) of age 6-8 weeks were obtained from Charles River Japan (Atsugi, Japan). Mice were anesthetized with pentobarbital sodium (60 mg/kg intraperitoneally) and injected intracerebrally with T98G cells (1 × 10^5^) through a small hole drilled 2 mm anterior and 2 mm lateral to the bregma. Immediately after cell implantation, MMI-166 (100 mg/kg), suspended in a vehicle (0.9% NaCl solution containing 0.5% carboxymethylcellulose Na, 0.9% benzyl alcohol, and 0.4% Tween 20) was orally administered to 10 mice five times a week for up to 21 days. The control group (n = 10) were orally administered with the vehicle alone. All mice were sacrificed on day 22, and their brains were snap-frozen. Tumour growth of the intracerebral tumours was confirmed by histological evaluation. Serial coronal sections (30 μm) were cut from the rostal to caudal edge of the brain tissues containing the tumours by using a cryo-microtome. Tumour size was computed using an Imagepro system (Media Cybernetics Inc., Silver Spring, MD, USA).

For immunohistological examination of angiogenesis, the same T98G xenograft model was used. Mice from both the groups were sacrificed on day 22, and their brains were harvested and fixed in buffered formalin before embedding in paraffin. Tumour angiogenesis was evaluated using Von Willebrand Factor (VWF) immunostaining. Immunohistochemistry was performed with primary antibodies specific for VWF (ab6994; Abcam Inc., Cambridge, MA, USA). Briefly, 4-μm-thick paraffin sections were deparaffinized and dehydrated. The sections were first incubated with appropriate primary antibodies and then with the EnVision^+ ^System HRP (DAKO, Glostrup, Denmark). Positive staining was detected using diaminobenzidine. For negative controls, the primary antibodies were replaced by a non-specific IgG. All procedures involving animals were approved by the animal care committee of Kochi University and were in accordance with institutional guidelines and Japanese government regulations.

### Statistical analysis

The invasion and angiogenesis assay results obtained from the control and treated groups were statistically analysed by one-way analysis of variance (ANOVA). The statistical significance of tumour volume between the control and treated groups in orthotropic implantation model was analysed using unpaired/paired Student's t-test. In all statistical analyses, p < 0.05 was regarded as statistically significant. All values are presented as means ± standard error (SE).

## Results

### Zymography

**Figure 3:**
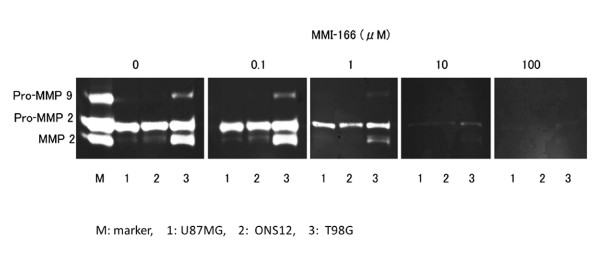
**Gelatine zymography**. The zymographic analysis revealed that the gelatinolytic activities of MMP-2 and MMP-9 decreased in a dose-dependent manner after the addition of MMI-166. Although MMP-9 activity was completely controlled by 10 μM MMI-166, MMP-2 activity remained and was mostly controlled by 100 μM MMI-166.

The effects of MMI-166 on the gelatinolytic activities of glioma cells were determined by gelatine zymography. The culture supernatants of glioma cell lines were assayed for MMP-2 and MMP-9 gelatinase activities. The glioma cells showed MMP-2 and MMP-9 gelatinase activities. Both the latent and active forms of MMP-2 and the latent form of MMP-9 were detected in the culture supernatant from glioma cells. We found that MMI-166 reduced the gelatinolytic activities of MMP-2 and MMP-9 in a dose-dependent fashion (Figure 3). Although MMP-9 activity was completely controlled by 10 μM MMI-166, MMP-2 activity remained and was mostly controlled by 100 μM MMI-166.

### Invasion assay

**Figure 4:**
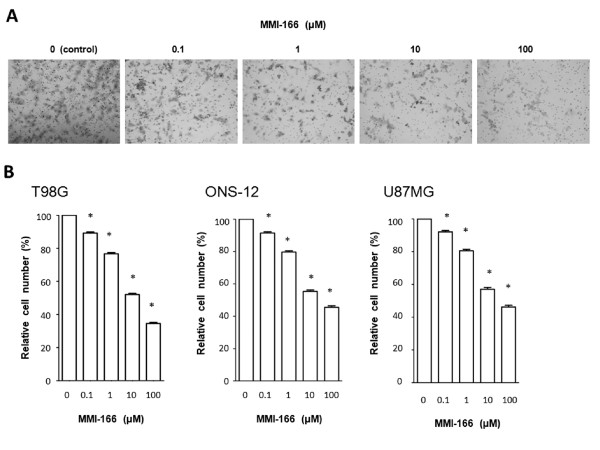
**Invasion assay**. The shown photographs are representative data of invasion assay of T98G (**A**). The number of invading glioma cells was compared with the control (non-treated). The number of invading cells decreased as the concentration of MMI-166 increased. The number of invading cells decreased as the concentration of MMI-166 increased in all three glioma cell lines (**B**). Statistically significant differences were observed between the MMI-166-treated samples and the control (non-treated) (*p < 0.0001).

The inhibitory effect of MMI-166 was evaluated by the in vitro invasion assay. MMI-166 at various concentrations was added to the medium. Figure 4A shows representative data of T98G. The number of invading glioma cells was compared with the control. The number of invading cells decreased as the concentration of MMI-166 increased. The average number of invading tumour cells in the three glioma cell lines decreased to 55.3 ± 0.91% and 45.6 ± 0.93%, after the addition of 10 and 100 μM MMI-166, respectively, compared to the control (Figure 4B). Statistical analysis revealed that invasion of glioma cells was significantly suppressed by MMI-166 (p < 0.0001).

### Angiogenesis assay

**Figure 5:**
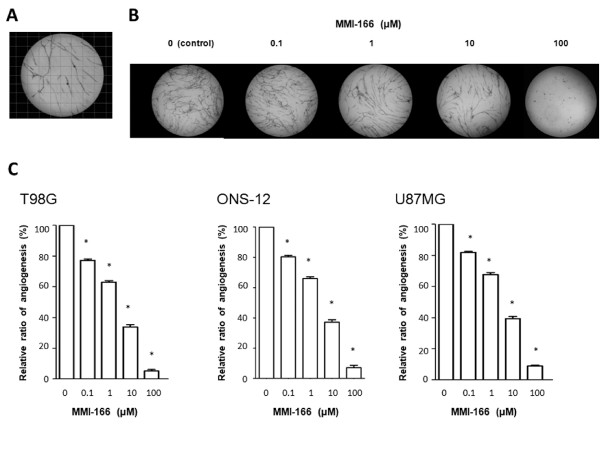
**Angiogenesis assay**. A blood vessel construction image was overlaid with a grid image of the same size, and neovascularity was evaluated as the total number of intersections between the vessel and grid in the entire plate (**A**). The shown photographs are representative data of invasion assay of T98G. The density of neovasculature decreased as the concentration of MMI-166 increased (**B**). The neovasculature induced by glioma cells decreased as the concentration of MMI-166 increased in all three glioma cell lines (**C**). Statistically significant differences were observed between the MMI-166-treated samples and the control (non-treated) (*p < 0.0001).

The inhibitory effect of MMI-166 on glioma-induced neovascularity was evaluated by an in vitro angiogenesis assay. A blood vessel construction image of the entire culture plate was captured by an image scanner. Subsequently, this image was overlaid with a grid image of the same size. The neovascularity was evaluated as the total number of intersections of the vessel and grid in the entire plate (Figure 5A). Figure 5B shows representative data for T98G. The density of neovasculature decreased with an increase in the concentration of MMI-166. The average total number of intersections of the vessel and grid in three glioma cell lines decreased to 37.2 ± 1.54% and 6.56 ± 0.70% after the addition of 10 and 100 μM MMI-166, respectively, when compared with the control (Figure 5C). Statistical analysis revealed that glioma cell-induced neovasularity was significantly suppressed by MMI-166.

### MTT assay

**Figure 6:**
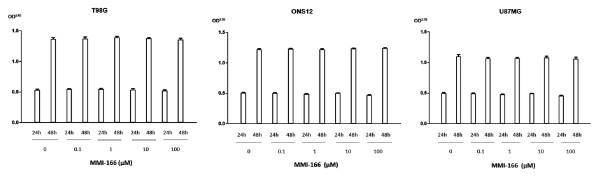
**MTT assay**. MMI-166 showed no inhibitory effect on the three glioma cell lines in the 2-day incubation period, up to a maximum concentration of 100 μM.

We assessed the inhibitory effect of MMI-166 on growing cultures of the three glioma cell lines by the MTT assay. During the 2-day incubation period, MMI-166 did not show any inhibitory effect on the growth of glioma cells (Figure 6).

### Evaluation of implanted tumours

**Figure 7:**
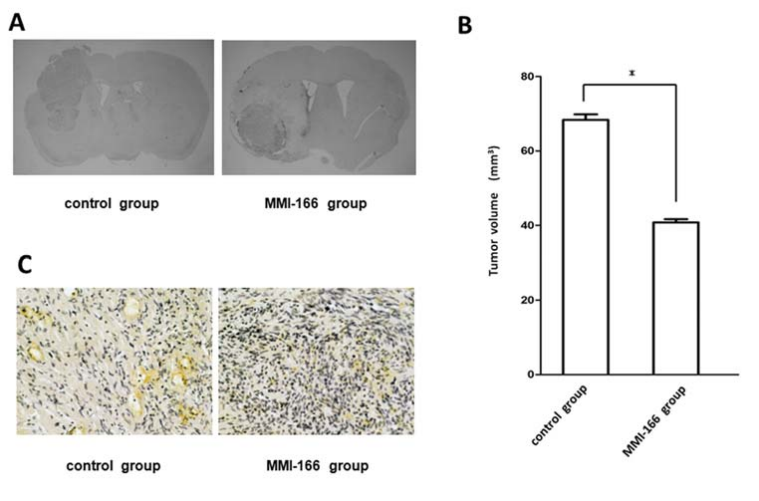
**Evaluation of implanted tumours**. The shown photographs are representative data of coronal sections of T98G xenografts (**A**). Quantitative analysis of tumour volume showed that the size of the transplanted tumours in the mice from the MMI-166 treatment group was significantly smaller than the transplanted tumour of the mice from the control group (* p < 0.0001, n = 10) (**B**). The shown photographs are representative sections of immunohistochemical staining for VWF (**C**). The expression of VWF was lower in the implanted tumour of the MMI-166 treatment group than in the implanted tumour of the control group.

In the MMI-166 treatment group (n = 10), tumour growth was suppressed compared to the control group (n = 10). The average tumour volume was calculated from sequential histological sections (Figure 7A), and was estimated to be 40.8 ± 0.87 mm^3 ^for the MMI-166 treatment group and 68.4 ± 1.45 mm^3 ^for the control group (p < 0.0001) (Figure 7B). Immunohistochemical analysis also showed that the number of microvessels (VWF expression) was lower in the MMI-166 treatment group than in the control group (Figure 7C). Histological studies of T98G xenograft model showed that MMI-166 inhibited tumour growth and angiogenesis in vivo.

## Discussion

Tumour growth includes increased synthesis and secretion of several proteases such as cysteine protease, serine protease, and MMP to degrade the ECM. These proteases participate in establishing and maintaining a microenvironment around the tumour so that the tumour cells can survive. Proteolytic action on the ECM is a key step required for the initiation of tumour invasion and angiogenesis. Recent studies suggest that angiogenesis and invasion cooperate in tumour development and involve similar biological mechanisms [7]. Furthermore, MMP is strongly related to tumour progression.

MMPs are a family of zinc endopeptidases comprising at least 20 different members. They are classified into five groups on the basis of their structure and substrate specificities [8]. MMPs are secreted as proenzymes that are activated after a peptide of approximately 10 kDa is cleaved. The activity of MMPs is primarily regulated at the transcriptional and translational levels by the secretion by hormones, growth factors, and cytokines [9]. Moreover, there is evidence regarding the modulation of mRNA stability in response to growth factors and cytokines [7,10]. Additionally, MMPs are inhibited by specific tissue inhibitor matrix metalloproteinases (TIMPs) and metal chelators [11].

The increased expression of MMPs has been associated with cancers of the head and neck, breast, lung, stomach, and pancreas [12-16]. In particular, MMP-2 and MMP-9 were reported to correlate with tumour grade and metastasis [17]. On the basis of the evidence that TIMPs can interfere with experimental metastasis, the role of MMPs in tumour progression has been determined [18]. However, the role of MMPs and TIMPs in cancer is much more complicated than that suggested initially. For example, increased TIMP-1 levels in human cancer tissues have been associated with poor prognoses [19]. It is uncertain whether this reflects growth-potentiating properties or some other undetermined property of TIMPs [20]. Other experimental studies involving cancer cells transfected with TIMP-1 cDNA demonstrated that MMPs act primarily to alter the extracellular environment to allow sustained cancer cell growth at an ectopic site as opposed to having the specific role of allowing the cells to extravasate from the blood stream [21]. Furthermore, in some experimental tumour systems, increased MMP production did not correlate with increased metastasis [22]. One possible explanation for this finding is that excess proteolysis might degrade matrix signals and receptors, thereby disrupting cell matrix interactions and inhibiting migration [23].

One of the early events in the transition of a tumour from the pre-neoplastic to the neoplastic state is the ability of the tumour to promote angiogenesis [24]. The results of numerous experimental studies support the concept that the growth of new blood vessels is required for continued tumour growth [25]. Tumour angiogenesis is a complex process that requires 1) the degradation of the basement membrane and ECM surrounding the blood vessels, 2) chemotaxis of endothelial cells towards an angiogenic stimulus, 3) proliferation of the endothelial cells, and 4) remodelling of the basement membrane as new blood vessels form. This remodelling is considered to result from MMP activity [26]. Endothelial cells produce MMP-1, MMP-2, MMP-3, and membrane-type MMP (MT-MMP). Recent studies revealed that MMP-2 and MT-MMP have a crucial role in angiogenesis (2). The treatment of human umbilical vein endothelial cells with phorbol ester (namely, phorbol 12-myristate 13-acetate; PMA) leads to MMP-2 activation and induction of MT-MMP [27]. This is accompanied by the formation of multicellular tube structures when cells are cultured in a collagen gel [28].

Malignant gliomas are highly aggressive tumours characterized by extensive brain invasion and strong angiogenesis. Recent proteinase profiling studies have demonstrated the over-expression of the serine urokinase-type plasminogen activator (uPA) and its receptor (uPAR), cysteine protease cathepsin B, MMP-2, and MMP-9 in high grade astrocytomas compared with low grade astrocytomas or the normal brain. In particular, MMP-2 and MMP-9 are the two most abundant MMPs found in gliomas [29]. Therefore, it has been proposed that MMP-2 and MMP-9 inhibitors can act as potential drugs for the treatment of gliomas. Selective gene suppression of MMP-2 or MMP-9 dramatically reduces the invasive phenotype of gliomas [30,31].

A number of MMP inhibitors, including Batimastat [3], Marimastat [4], and AG3340 [32], have entered clinical development, but none have been licensed. Disappointing results were reported for phase III studies in patients with non-small cell lung cancer stage IIIB/IV [33], metastatic breast cancer [34], and advanced pancreatic adenocarcinoma [35]. However, it is still thought that MMP inhibitors may have therapeutic potential for the earlier stages of cancer or prevention of cancer metastasis. Although MMP inhibitor has not cytotoxic anticancer effect, MMP inhibitor has cytostatic and anti-angiogenic effects. Furthermore, MMP inhibitor has few side effects as compared with cytotoxic anticancer drug and also has the merit of controlling a cancer more effectively when it was used together with other cytotoxic drugs.

As for glioma, AG3340 and SI-27 [36] were preclinically studied. In vivo study using SCID-NOD mouse, AG3340 decreased tumour size of transplanted U87 glioma by 78% compared with controls after 31 days [37]. Systemic administration of SI-27 in U251MG xenograft mouse showed a statistically significant increase in survival time compared with the controls receiving carrier (median survival, 47.3 versus 32.6 d). There was also a decrease in MMP activity, tumour cell invasion, and neovascularization [36]. Therefore, it is thought MMP inhibitors may have therapeutic potential for gliomas.

MMI-166 is a third generation MMP inhibitor having an *N*-arylsulfonyl-*α*- aminocarboxylate zinc binding group. It is an MMP-2, -9 and -14 selective inhibitor that spares MMP-1, -3 and -7. The key structural feature exemplified by MMI-166 is the "deep" aryl substitution. While it has shown anticancer activity in several animal models of human cancer, there are no data as to its effect on malignant gliomas. Therefore, we aimed to examine the effect of MMI-166 on malignant glioma cells.

So for, there is no data about the clinical trial of MMI-166. However, S-3304 (Shionogi & Co., Ltd; Osaka, Japan), a relative compound of MMI-166, has shown a good safety profile and good systemic exposure when administered orally in doses up to 800 mg twice daily for 10 to 17 days in healthy volunteers [41]. A phase I pharmacokinetic and pharmacodynamic study of S-3304 in patients with advanced and refractory solid tumours showed that S-3304 is safe, well tolerated, and achieves plasma concentrations above those required to inhibit MMP-2 and MMP-9 [42].

In the present study, we used a new in vitro angiogenesis assay. Tumour-induced angiogenesis is usually performed by the dorsal air sac-chamber assay or the chick choriallantoic membrane assay (CAM assay). However, these two methods need animals and are slightly complicated. An in vitro angiogenesis assay that does not require an animal is usually carried out by adding the tumour culture supernatant to an angiogenesis kit in which the endothelial cells were set. However, there is no direct traffic between the endothelial and tumour cells in this technique. In contrast, our new method enables direct trafficking between the endothelial and tumour cells through secreting factors from each cell type.

## Conclusion

The present study showed that MMI-166 reduced the activities of MMP-2 and MMP-9 and significantly inhibited the invasive and angiogenic activities of glioma cells in vitro and in vivo. Furthermore, MMI-166 inhibited tumour growth in vivo. It is possible that MMI-166 potentiates the suppression of glioma progression.

## Competing interests

The authors declare that they have no competing interests.

## Authors' contributions

HN was responsible for the study design, interpretation of the data and revision of the manuscript. TY and KS supervised the studies and helped to revise the manuscript. All readers read and approved the final manuscript.

## Pre-publication history

The pre-publication history for this paper can be accessed here:

http://www.biomedcentral.com/1471-2407/10/339/prepub
